# Carotid intima-media thickness, fibroblast growth factor 23, and mineral bone disorder in children with chronic kidney disease

**DOI:** 10.1186/s12882-024-03771-z

**Published:** 2024-10-21

**Authors:** Retno Palupi-Baroto, Kristia Hermawan, Indah Kartika Murni, Tiara Nurlita, Yuli Prihastuti, Ira Puspitawati, Chika Carnation Tandri, Cahyani Gita Ambarsari

**Affiliations:** 1 Division of Nephrology, Department of Child Health, Faculty of Medicine, Public Health and Nursing Universitas Gadjah Mada - Dr Sardjito Hospital, Jl. Farmako Sekip Utara, Yogyakarta, 55281 Indonesia; 2 Division of Cardiology, Department of Child Health, Faculty of Medicine, Public Health and Nursing Universitas Gadjah Mada - Dr Sardjito Hospital, Yogyakarta, Indonesia; 3 Department of Child Health, Faculty of Medicine, Public Health and Nursing Universitas Gadjah Mada, Yogyakarta, Indonesia; 4 Clinical Pathology and Laboratorium Medicine, Faculty of Medicine, Public Health and Nursing Universitas Gadjah Mada - Dr Sardjito Hospital, Yogyakarta, Indonesia; 5 Faculty of Medicine Universitas Indonesia - Cipto Mangunkusumo Hospital, Jakarta, Indonesia; 6 Department of Child Health, Faculty of Medicine Universitas Indonesia - Cipto Mangunkusumo Hospital, Jakarta, Indonesia; 7 School of Medicine, University of Nottingham, Nottingham, UK; 8 Medical Technology Cluster, Indonesian Medical Education and Research Institute (IMERI), Faculty of Medicine Universitas Indonesia, Jakarta, Indonesia

**Keywords:** Calcium, Dialysis, End-stage kidney disease, Hypertension, Left ventricular hypertrophy, Parathyroid hormone, Phosphorus

## Abstract

**Background:**

Carotid intima-media thickness (cIMT) is a measure of atherosclerotic vascular disease and a surrogate biomarker for cardiovascular risk in patients with chronic kidney disease (CKD). Mineral and bone disorders (MBD) are complications of CKD, contributing to vascular calcification and accelerated atherosclerosis. Increased fibroblast growth factor 23 (FGF23)—the earliest detectable serum abnormality associated with CKD-MBD—has been linked with cardiovascular disease in patients with CKD. This study aimed to identify factors and analyze the relationship associated with high cIMT, high FGF23, and poor MBD control in children with CKD.

**Methods:**

A cross-sectional study was conducted in Yogyakarta, Indonesia recruiting children with CKD. The correlations and factors between cIMT, FGF23, and MBD were explored.

**Results:**

We recruited 42 children aged 2–18 years old with CKD stages 2 to 5D. There were no significant correlations between cIMT and factors including advanced CKD, use of dialysis, body mass index, hypertension, anemia, MBD, FGF23 levels, and left ventricular mass index (LVMI). Patients with advanced CKD had poorly controlled anemia, hypertension, and higher LVMI. In multivariate analysis, CKD stages, hypertension stages, the presence of MBD, and LVMI were associated with FGF23 levels (*p* < 0.05).

**Conclusions:**

FGF23 levels increased with CKD progression, and MBD was more prevalent in advanced kidney disease. Elevated FGF23 is potentially associated with increased MBD prevalence in late-stage CKD. A larger study is needed to confirm the factors affecting cIMT in children with CKD.

**Supplementary Information:**

The online version contains supplementary material available at 10.1186/s12882-024-03771-z.

## Introduction

The kidneys are key organs for the regulation of bone and mineral metabolism [1]. Chronic kidney disease (CKD) leads to various metabolic conditions, with mineral and bone disorders (MBD) being the most common. CKD-MBD is characterized by aberrant calcium, phosphate, parathyroid hormone (PTH), and vitamin D metabolism, dysfunctional bone turnover with disturbances in osteoclast/osteoblast balance (renal osteodystrophy), and vascular calcification [2]. CKD-MBD can cause bone pain, deformities, and fractures that impact the daily lives and well-being of children with advanced CKD. These issues persist into adulthood, with up to 18% of affected individuals experiencing bone disease-related disability [3]. CKD-MBD disrupts the mineral and bone axis, leading to vascular calcification and accelerated atherosclerosis through local inflammation, elastin degradation, and osteogenic differentiation of vascular smooth muscle [4]. Children with CKD, especially those undergoing dialysis, face significantly higher risks of cardiovascular morbidity and mortality and vascular calcifications are evident in children and young adults with end-stage kidney disease (ESKD). Effective CKD-MBD management during childhood is essential to optimize bone health and prevent cardiovascular disease progression [2].

Carotid intima-media thickness (cIMT) is a non-invasive ultrasound examination measuring the combined thickness of the inner carotid artery layers. Thickening is an early indicator of potential atherosclerotic vascular disease since it is apparent even when patients are asymptomatic [5]. Consequently, cIMT can be used as a biomarker for subclinical and asymptomatic atherosclerotic vascular disease and other cardiovascular disease in patients with CKD. Early cIMT measurement helps identify patients who may benefit from more intensive therapy to minimize disease progression and thus improve outcomes [6]. However, studies in children with CKD have yielded conflicting results regarding the correlation between cIMT and kidney function. Although increased cIMT has been noted in pre-dialysis CKD patients, no correlations have been found between increased cIMT and decreased eGFR or CKD progression [7–9].

Fibroblast growth factor 23 (FGF23) is a hormone produced by osteocytes and osteoblasts; it plays a crucial role in regulating phosphorus and vitamin D levels in the kidneys and bones [10]. Our previous study in children with CKD stages 2 to 5D showed that FGF23 levels increased as early as CKD stage 2 and were correlated with left ventricular hypertrophy and severe cardiac impairment [11, 12]. In these children, FGF23 rises with worsening kidney function but is preceded by increases in parathyroid hormone (PTH) and phosphate levels [11]. Reduced renal phosphorus excretion leads to a reduction in 1,25-dihydroxyvitamin D due to 1-hydroxylase inhibition [13]. FGF23 and PTH jointly regulate calcium reabsorption in the kidneys. Accordingly, low calcium levels and secondary parathyroidism may contribute to elevated FGF23 levels [14]. Research has also demonstrated a connection between uric acid and vascular calcification, which is associated with cIMT. Additionally, uric acid suppresses the 1 α-hydroxylase enzyme, resulting in reduced 1,25-dihydroxyvitamin D (1,25(OH)2D) levels and elevated intact parathyroid hormone (iPTH) levels [4].

**Fig. 1:**
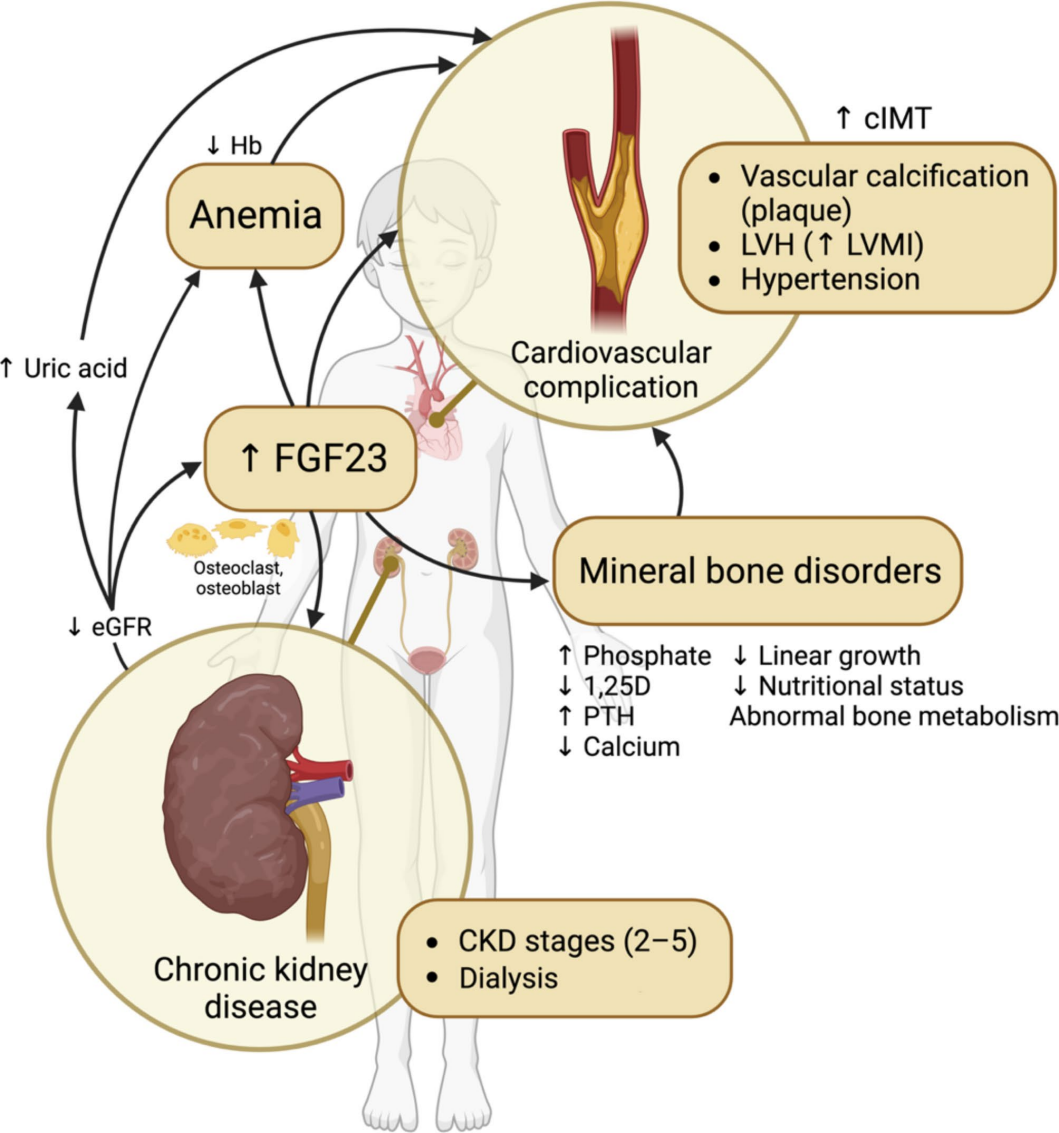
Hypothesized relationship between MBD, FGF23, and cardiovascular outcomes. Created with BioRender.com. 1,25D: 1,25-dihydroxyvitamin D; cIMT: carotid intima-media thickness; CKD: chronic kidney disease; eGFR: estimated glomerular filtration rate (mL/min per 1.73 m^2^); FGF23: fibroblast growth factor 23; Hb: hemoglobin; LVH: left ventricular hypertrophy; LVMI: left ventricular mass index; MBD: mineral bone disorders; PTH: parathyroid hormone

Recent research suggests that FGF23 may be involved in CKD-MBD development [15, 16]. Although the relationship between high FGF23 levels and CKD-MBD has been described in children [17–21], obtaining a better understanding of the relationship between cIMT, FGF23, and MBD in children with CKD may help improve disease management. Some studies have yielded contradictory results. Singh et al. (2022) found increased FGF23 levels with higher CKD grades; however, no significant association was present between FGF23 and cardiovascular parameters, including cIMT [22]. Preka et al. (2018) found no associations between cardiovascular markers indicating early arterial damage, such as cIMT, and pediatric CKD patients’ biochemical or bone data [23]. These conflicting findings suggest that the relationship between FGF23, cIMT, and MBD is yet to be confirmed. This study aimed to (i) identify factors associated with high cIMT, high FGF23, and poor MBD control, and (ii) analyze the relationships between cIMT, FGF23, and MBD in children with CKD. Our hypothesis regarding these relationships is presented in Fig. 1.

## Methods

### Participants

This cross-sectional study included pediatric patients with CKD stages 2 to 5D in Dr. Sardjito General Hospital, Yogyakarta, Indonesia, a regional referral hospital for Yogyakarta and southern Central Java, between November 2018 and March 2019. Inclusion criteria were children aged 2 to 18 years diagnosed with CKD stages 2 to 5D based on the Kidney Disease Improving Global Outcomes (KDIGO) criteria [24, 25]. Patients with congenital heart disease, acquired heart disease (e.g., rheumatic heart disease, Kawasaki disease, and myocarditis), diabetic nephropathy, or kidney malignancy were excluded.

The study was approved by the Dr. Sardjito General Hospital Institutional Review Board and the Medical and Health Research Ethics Committee Faculty of Medicine, Public Health and Nursing Universitas Gadjah Mada (KE/FK/1251/EC/2018). It was conducted in accordance with the Declaration of Helsinki. All patients’ caregivers provided written informed consent. Demographic and clinical data were obtained by the doctors and nurses during an outpatient visit or hospital admission.

### Blood pressure measurement

Blood pressure was measured by a nurse according to the 2017 American Academy of Pediatrics Blood Pressure Clinical Practice Guideline [26]. The average of three measurements was used and results were categorized accordingly (Table S1) [26].

### Plasma FGF23 measurement

Venous blood samples were collected in Ethylene Diamine Tetra Acetic (EDTA)-containing vacutainer tubes. Plasma FGF23 was measured by quantitative sandwich enzyme-linked immunosorbent assay (ELISA). A specific FGF23 carboxyl-terminal (C-terminal) kit was obtained from Immutopic, Inc. (San Clemente, CA 92673, USA), and FGF23 levels were determined by immunometric enzyme assay using a Biorad 680 Microplate Reader and Microplate Manager software version 5.2.1 (both from Bio-Rad Laboratories Inc., CA, USA). Three milliliters of the venous blood in each EDTA tube were taken and centrifuged into plasma within 2 h. Results were expressed in RU/mL and calibration standards ranged from 21 RU/mL to 1509 RU/ml. The normal range of FGF23 was based on a previous study [27].

### Measurement of estimated glomerular filtration rate

Estimated glomerular filtration rate (eGFR) was calculated using the revised Schwartz Formula (36.5 x L/Cr, where L represents body length in centimeters, and Cr represents serum creatinine concentration in µmol/L [28]). GFR was expressed as mL/min per 1.73 m^2^ body surface area. CKD stages were based on the KDIGO 2023 Clinical Practice Guideline for the Evaluation and Management of Chronic Kidney Disease [24, 25]. We categorized CKD based on etiology as glomerular disease (steroid-resistant nephrotic syndrome, post-infectious glomerulonephritis, and lupus nephritis), congenital anomalies of kidney and urinary tract (CAKUT; obstructive uropathy, kidney aplasia/hypoplasia/dysplasia, and reflux nephropathy), and others (polycystic kidney disease [PKD], tubular disorders, and nephrocalcinosis).

### Measurement of cIMT

CIMT is the distance between the intima and media layers of the carotid artery as measured by vascular echocardiography [29]. Measurement was performed using a Philips HD15 echocardiogram machine using ultrasound mode and vascular Doppler by one examiner who was blind to patients’ CKD stage and laboratory data. The patient was in a supine position with the neck rotated to the left. The probe was positioned horizontally on the lateral right side of the neck, aligned with the thyroid cartilage. After the far wall of the carotid artery was visualized, 1–2 cm of the proximal side of the carotid artery bifurcation was identified to visualize the common carotid artery segment. The area was scanned using anterior and posterior projections, and then the border between the media and adventitia layers to the border between the intima layer and the vascular lumen was measured manually using calipers [30]. All measurements were recorded in millimeters and stored digitally for analysis. The normal range of cIMT differs based on gender, age, and body mass index (BMI) [31, 32]. To classify the measured cIMT, we utilized the percentile table proposed by Doyon et al. (2013), categorizing measurements exceeding the 95th percentile as indicative of an increased cIMT [32]. We also assessed left ventricular mass by echocardiography and calculated left ventricular mass index (LVMI) by dividing left ventricle mass by body surface area (g/m^2^) [33].

### Blood chemistry

Blood samples were drawn during the study to measure FGF23 as well as hemoglobin, creatinine, calcium, phosphorus, and PTH. Normal hemoglobin was defined according to KDIGO and WHO criteria [34, 35]. CKD-MBD was defined as a systemic disorder of mineral and bone metabolism due to CKD manifested by at least one of the following: (i) abnormalities of calcium, phosphorus, PTH, or vitamin D metabolism; (ii) abnormalities in bone turnover, mineralization, volume, linear growth, or strength; and (iii) vascular or other soft tissue calcification [36, 37]. Normal ranges for parameters associated with MBD are presented in Tables S2–S4 [38, 39].

### Statistical analysis

Categorical variables were expressed as percentages, and medians with interquartile ranges (IQR) were used for non-normal data. Laboratory and clinical characteristics were compared using the Spearman’s Rank correlation test for numeric data. Mann-Whitney U tests were used to assess differences between two independent groups, while Kruskal-Wallis tests were utilized for comparisons involving more than two groups. *P* values of less than 0.05 (*p* < 0.05) were considered to be statistically significant. We conducted a multivariate regression analysis, selecting four variables for our analysis given their clinical importance and to avoid multicollinearity. All statistical analyses were performed using Stata/BE 18 software.

## Results

### Study population

Baseline characteristics of the 42 children with CKD stages 2 to 5D included in the study are listed in Table 1. Of the 42, 22 (52.38%) were female. The median age was 12.7 (9.3–15.4) years when FGF23 was tested. Patients had a median BMI of 17 (14.51–22.03) kg/m^2^ and a height standard deviation score of -2.35 (-3.65 to -1.53). Most patients had a primary CKD diagnosis of glomerular disease (20/42, 47.62%), while 18/42, 42.86% had CAKUT, and 4/42, 9.52% had other disorders. Glomerular diseases included nephrotic syndrome (12/42, 28.57%), lupus nephritis (2/42, 4.65%), and other glomerulonephritis (6/42, 14.29%). CAKUT disorders included obstructive uropathy (7/42, 16.67%), kidney aplasia/hypoplasia/dysplasia (10/42, 23.81%), and reflux nephropathy (1/42, 2.38%). Other disorders included PKD (3/42, 7.14%) and bilateral nephrocalcinosis (1/42, 2.38%) (Table S5). Most patients (32/42, 76.19%) had MBD (details shown in Table 1 and S6). FGF23 levels were highly variable, with a median of 723.56 (264.88–1493.2) RU/mL. In contrast, cIMT was similar among the patients, with a median of 0.42 (0.4–0.45) mm. Additionally, median of dialysis duration was 16.39 (6.48–28.87) months.

**Table 1:** Baseline characteristics of the study population

Characteristics	Overall	CKD Stages			*p* value	
		Stages 2–3	Stages 4 and 5ND	Stage 5D		
Number of patients	42	12	10	20		
**Demographic**						
Age as a subject in our study, years	12.7 (9.3–15.4)	12.9 (9.6–15.4)	14.2 (9.3–16.1)	12.1 (9.7–14.6)	NS	
Sex, female	22 (52.38)	7 (58.33)	5 (50)	10 (50)	NS	
BMI^†^, kg/m^2^	17 (14.51–22.03)	17.91 (16.01–23.66)	14.94 (13.53–23.31)	17.41 (14.85–18.7)	NS	
**Primary diagnosis of CKD**						NS
Glomerular diseases^‡^, n (%)	20 (47.62)	8 (66.67)	3 (30)	9 (45)		
CAKUT disorders^§^, n (%)	18 (42.86)	4 (33.33)	5 (50)	9 (45)		
Others^||^, n (%)	4 (9.52)	0	2 (20)	2 (10)		
**CKD complications**						
Height SDS^*^	-2.35 (-3.65 – -1.53)	-3.25 (-5.17 – -1.91)	-2.3 (-3.22 – -1.53)	-2.20 (-2.99 – -1.54)	NS	
Anemia, n (%)	32 (76.19)	6 (50)	8 (80)	18 (90)	0.035	
Hypertension, n (%)	23 (53.49)	4 (33.33)	4 (40)	14 (70)	NS	
Mineral bone disorders, n (%)	32 (76.19)	6 (50)	9 (90)	17 (85)	0.04	
**Serum biochemistry**						
Calcium (mg/dL)	9.34 (8.34–9.94)	9.34 (9.06–9.68)	9.34 (8.34–9.98)	9.02 (7.88–10.4)	NS	
Phosphate (mg/dL)	3.89 (3–5.2)	3.8 (3.35–4.7)	5 (3.7–5.8)	3.65 (2.65–5.1)	NS	
Ca x P product (mg^2^/dL^2^)	35.72 (24.46–51.1)	35.72 (29.19–41.43)	48.95 (30.63–55.62)	31.92 (23.09–51.32)	NS	
FGF23 (RU/mL)	723.56 (264.88–1493.2)	281.26 (178.05–415.8)	1029.54 (254.83–1390.07)	1350.13 (583.91–6037.9)	0.001	
PTH (pg/mol)^†^	232.55 (113.3–489.5)	232.9 (112.05–503.8)	168.1 (117.6–489.5)	297 (113.3–478)	NS	
**Echocardiography**						
cIMT (mm)	0.42 (0.4–0.45)	0.42 (0.37–0.45)	0.4 (0.39–0.42)	0.43 (0.4–0.49)	NS	
LVMI (g/m^2^)	64.42 (52.38–115.05)	53.79 (48.53–62.66)	65.64 (52.66–90.45)	115.27 (55.84–151.11)	0.014	

BMI: body mass index; CAKUT: congenital anomalies of the kidney and urinary tract; Ca: Calcium; cIMT: carotid intima-media thickness; CKD: chronic kidney disease; CKD 5ND: eGFR < 15 mL/min per 1.73 m^2^ not on dialysis; CKD 5D: eGFR < 15 mL/min per 1.73 m^2^ with dialysis; FGF23; fibroblast growth factor 23; LVMI: left ventricular mass index; N/A: not applicable; NS: not significant; SDS: standard deviation score

^*^Based on Centers for Disease Control and Prevention (CDC) Growth Chart [40]

^†^BMI: underweight (BMI-for-age < 5th percentile), normal (BMI-for-age ≥ 5th and < 85th percentile), overweight (BMI-for-age ≥ 85th and < 95th percentile), obese (BMI-for-age ≥ 95th percentile) [40]

^‡^Glomerular diseases: nephrotic syndrome, post-infectious glomerulonephritis, lupus nephritis

^§^CAKUT: obstructive uropathy, reflux nephropathy, and kidney aplasia/hypoplasia/dysplasia

^||^Others: polycystic kidney disease and nephrocalcinosis due to Williams syndrome

^†^PTH data were from 18 subjects: stage 2–3 (*n* = 4), stage 4–5ND (*n* = 5), stage 5D (*n* = 9)

### Factors associated with cIMT and plasma FGF23 in pediatric patients with CKD

We investigated associations between cIMT and MBD parameters, cardiovascular measurement, and other factors, and between plasma FGF23 and the same factors (Table 2, S7). In univariate analysis, we found a significant positive correlation between FGF23 and LVMI (*r* = 0.5, *p* < 0.001). Furthermore, Mann-Whitney tests demonstrated that FGF23 was significantly correlated with the presence of MBD (*p* < 0.01) as well as kidney replacement therapy (KRT) (*p* < 0.01). Kruskal-Wallis analysis showed that FGF23 was significantly correlated with CKD stage (*p* < 0.01) and hypertension (*p* < 0.02). No variables were significantly correlated with cIMT. Further multivariate regression analysis showed that LVMI, CKD stages, hypertension stages, and the presence of MBD were associated with FGF23 levels and no variables were found to be associated with cIMT (Table 3).

**Table 2:** Correlations of cIMT and FGF23 with MBD, cardiovascular parameters, and other CKD variables^*^

Variables	cIMT		FGF23	
	*r*	*p*	*r*	*p*
**Demographic**				
Age (years)*	0.21	NS (0.42)	-0.1	NS (0.5)
Sex**	0.26	0.25	0.13	0.56
BMI (kg/m^2^)*	0.13	NS (0.81)	-0.09	NS (0.57)
**Primary diagnosis of CKD**				
Primary disease (CAKUT, glomerular, and others)***	0.01	0.79	0.01	0.77
**CKD complications**				
Stunted**	0.18	0.52	0.13	0.63
Anemia**	0.23	0.47	0.56	0.08
Hypertension**	0.01	0.98	0.35	0.11
Mineral bone disorders**	0.36	0.26	0.81	< 0.01
Receiving KRT^†**^	0.32	0.16	0.7	< 0.01
CKD stage***	0.1	0.51	0.38	< 0.01
**Serum biochemistry**				
Calcium (mg/dL)*	-0.002	NS (0.99)	0.08	NS (0.6)
Phosphate (mg/dL)*	0.06	NS (0.69)	0.1	NS (0.52)
Ca x P product (mg^2^/dL^2^)*	0.05	NS (0.73)	0.19	NS (0.23)
FGF23 (RU/mL)*	0.05	NS (0.73)	NA	NA
PTH (pg/mol)^‡*^	0.19	NS (0.46)	0.33	NS (0.18)
**Echocardiography**				
LVMI (g/m^2^)*	-0.01	NS (0.19)	0.5	< 0.001
Dialysis duration (months)	0.02	NS (0.92)	0.26	NS (0.27)

BMI: body mass index; CAKUT: congenital anomalies of kidney and urinary tract; Ca: Calcium; cIMT: P: phosphate; carotid intima-media thickness; KRT: kidney replacement therapy; CKD: chronic kidney disease; eGFR: estimated glomerular filtration rate (mL/min per 1.73 m^2^); FGF23: fibroblast growth factor 23; LVMI: left ventricular mass index; MBD: mineral bone disorders; PTH: parathyroid hormone; NS: not significant

^*^Spearman’s rank correlations

^**^Mann-Whitney tests

^***^Kruskal-Wallis tests

^†^KRT refers to hemodialysis and peritoneal dialysis

^‡^ PTH levels were tested in 18 subjects

^†^BMI: underweight (BMI-for-age < 5th percentile), normal (BMI-for-age ≥ 5th and < 85th percentile), overweight (BMI-for-age ≥ 85th and < 95th percentile), obese (BMI-for-age ≥ 95th percentile) [40]

^‡^categorized based on AAP, Table S1

**Table 3:** Multivariate analysis of factors associated with increased cIMT and FGF23 levels

Variables	cIMT		FGF23	
	*R* ^2^	*p* value	*R* ^2^	*p* value
CKD stages	0.05	0.15	0.19	0.004
Hypertension stages	0.01	0.55	0.12	0.03
Mineral bone disorders	0.11	0.04	0.11	0.03
LVMI (g/m^2^)	0.001	0.46	0.12	0.02

cIMT: carotid intima-media thickness; CKD: chronic kidney disease; FGF23: fibroblast growth factor 23; LVMI: left ventricular mass index

### Plasma FGF23 and bone mineral status in CKD stage 5 dialysis recipients

The median plasma FGF23 concentrations in children undergoing hemodialysis (HD) and peritoneal dialysis (PD) were 3986.47 (518.99–7326.01) RU/mL and 1350.13 (583.91–5153.06) RU/mL respectively (*p* = 0.78) (Table S8).

## Discussion

Our prior research, focusing on FGF23 as a biomarker for cardiac impairment, revealed cardiac changes in pediatric CKD patients, particularly highlighting left ventricular hypertrophy and impaired systolic function among those with CKD stage 2 and higher [12]. Meanwhile, our current study addresses aspects of the relationship between cIMT, FGF23, and MBD that we have yet to investigate. In this study, we explored the relationship of cIMT, high FGF23, and poor MBD in 42 children with CKD in Yogyakarta, Indonesia. The results of this study indicate a positive association of FGF23 levels with increased progression of kidney disease. Further, elevated FGF23 may be associated with the more prevalent MBD in the late stage of CKD.

Growth failure is a prevalent complication in children with CKD [2]. In this study, 57.14% of children with CKD were undernourished, and 69.05% had impaired growth. The etiology of growth impairment in children with CKD is multifactorial and includes both malnutrition and MBD [41]. The primary causes of kidney failure in children have been reported to be CAKUT (48–59%), glomerulonephritis (5–14%), hypertension (10–19%), hemolytic uremic syndrome (2–6%), cystic (5–9%), and ischemic nephropathy (2–4%) [2, 42–51]. Previously published data from a national referral center for pediatric dialysis in Indonesia suggested that 51.7–81.8% of pediatric patients receiving KRT had CAKUT [52, 53]. In this study, we found that most KRT was associated with glomerulonephritis and CAKUT, rather than other causes (45%, 45%, and 10%, respectively). We also observed significant increases in MBD and anemia as CKD progressed (*p* = 0.04 and 0.035, respectively).

We examined factors influencing cIMT, FGF23, and parameters related to MBD and explored their interrelationships. Our findings revealed significant associations between higher FGF23 levels and MBD, lower eGFR, more advanced CKD progression (particularly those requiring KRT), hypertension, and greater LVMI. The correlation with LVMI is in agreement with previous studies and increased FGF23 has been reported as a biomarker of myocardial hypertrophy in CKD [12, 54]. Anemia, hypertension, MBD, and malnutrition were prevalent among the study population, and higher FGF23 levels correlated with CKD severity and cardiovascular parameters, such as hypertension and LVMI, consistent with our hypothesis (Fig. 1). However, no significant correlation was observed between FGF23 and cIMT. Similar to earlier studies that failed to establish an association between cIMT and GFR or CKD stages [7–9, 55], none of the parameters in our study correlated significantly with cIMT (Tables 3, 4 and 5). This may have been caused by the uniformity of cIMT measurements we found across the CKD stages, and may be attributable to a few limitations in our cIMT measurement. Firstly, in our study, only one blinded examiner performed single anterior and posterior measurement, while several large-scale pediatric cIMT studies used averages from 5 to 6 consecutive measurements undertaken by three examiners [29, 31]. Although ultrasound-based cIMT measurement is safe, challenges include reproducibility and operator dependency [56, 57]. Secondly, to reduce the measurement error by a single reader, Peters et al. (2013) recommended having a batch reading, and performing the cIMT measurement at one point in time, because operator reading behavior may change over time [58]. However, this was not applicable in our setting because of geographical and financial reasons. Being a regional referral center, we had challenges in arranging for patients living remotely from our hospital to attend specific schedules for examinations. All of our patients had low socio-economic backgrounds, and therefore, their carers did not have the flexibility to come to our center to follow the planned schedule. Another important point to consider is that having healthy controls, lacking in our study, could be beneficial to better understand the cIMT characteristics in our CKD patients compared to the general pediatric population in our setting.

Schaefer et al. (2017) found that only 41.6% of children with CKD stages 3–5 had elevated cIMT, and 10.8% of patients had cIMT below the 50th percentile. Furthermore, no correlation between cIMT and eGFR was evident (*r* = − 0.06, *p* = 0.15) [7]. Results from the Chronic Kidney Disease in Children (CKiD) study also demonstrated that in children with mild to moderate CKD, there was only a − 0.002 (–0.01–0.001) mm mean difference in the cIMT measurement in every 10 mL/min per 1.73 m2 decrease in the eGFR, and there was no significant correlation between cIMT and eGFR [52]. In addition, Lopes et al. (2019) reported that although 74.5% of children with CKD stages 2–5 had elevated cIMT, the prevalence of increased cIMT value did not differ significantly across the CKD stages (*p* > 0.05) [9]. However, elevated cIMT correlated with hypertension, body fat percentage, and pubertal stage. Thus, we consider that cIMT might not change in children to the same extent as it does in the adult population with CKD [6, 59].

Portale et al. (2014) reported that levels of FGF23 were lowest in CKD stage 2, with a median of 93 (73–140) RU/mL, and increased as CKD progressed [60]. In our study, FGF23 levels were higher (Table 2) than those reported by Portale et al. at all stages [60], and increased substantially as kidney function declined. As reported in previous studies [61, 62], we also found that FGF23 levels in patients with CKD undergoing KRT were markedly elevated compared with CKD pre-dialysis. Rodello-Haad et al. (2018) reported a median FGF23 level of 900.5 (400.2–1819.7) RU/mL in 150 adult patients undergoing HD [61]. In pediatric patients on HD, Seeherunvong et al. (2012) reported that FGF23 levels can be extremely high, reaching up to 835-fold above the upper limit of normal (200 RU/mL) [62]. The poorly controlled MBD demonstrated by hyperphosphatemia as well as undernutrition in our study subjects may have contributed to this. Lower serum calcium, higher serum phosphate, decreased 1,25-dihydroxyvitamin D levels, and higher PTH levels further increase plasma FGF23 levels, resulting in extremely high FGF23 levels [63, 64]. Yamada et al. (2014) suggested that phosphate overload can induce systemic inflammation and malnutrition [65], and malnutrition has been reported to induce chronic inflammation, which also stimulates FGF23 production [66]. Additionally, late referral and delayed CKD diagnosis have been major issues in Indonesia, likely contributing to the notably elevated levels of FGF23 observed [67].

Our study found no significant differences in FGF23 levels between glomerular diseases, CAKUT disorders, and other CKD causes. Inconsistent with our findings, previous studies reported higher FGF23 levels in glomerular diseases than in non-glomerular diseases [60, 63]. FGF23 increases in glomerular diseases have been associated with corticosteroid use [63]. Prolonged corticosteroid administration may elevate FGF23 by inhibiting osteoblastic metabolism, followed by osteocyte activation and apoptosis. This sequence ultimately increases FGF23 synthesis, primarily by osteocytes [60]. During our study, 13.95% of children received corticosteroids for glomerular diseases; all received minimum dosages equivalent to prednisolone 5 mg daily. However, prior corticosteroid use and treatment duration were not documented. Another study reported contrasting results, showing that glucocorticoid treatment downregulated FGF23 and suppressed FGF23 synthesis [68]. Liu et al. (2023) reported that FGF23 levels were not associated with corticosteroid prescriptions, but were correlated to the degree of proteinuria in CKD due to primary nephrotic syndrome [69]. Our findings may have been affected by the uneven distribution of primary diseases, since 40% of cases related to glomerular disorders remained in CKD stages 2–3, while CAKUT and other etiologies predominantly contributed to ESKD.

We also analyzed the various parameters in patients receiving PD and HD and found no differences between the two modalities (Table S8). In our study, 85% of children with CKD stage 5 on long-term dialysis had MBD. According to the Kidney Disease Outcomes Quality Initiative, the target PTH range for children receiving dialysis is 150–300 pg/mL [35]. In our study, 41.6% of children had PTH > 300 pg/mL. Similar to our study, Bi et al. (2017) reported that PTH levels were higher in adults receiving HD than in those receiving PD [70]. Hyperphosphatemia, hypocalcemia, and low 1,25-dihydroxyvitamin D contribute to the development of secondary hyperparathyroidism in patients with CKD [8]. Overall, children receiving HD have more MBDs than those receiving PD [70]. However, we did not assess 1,25-dihydroxyvitamin D levels, use of active vitamin D analogs, cinacalcet, oral phosphate binders, and diets that can affect bone minerals in patients with CKD. In our setting, lack of laboratory testing for vitamin D, limited government support for vitamin D analogs, and the absence of cinacalcet in government hospital pharmacies make the diagnosis and treatment of CKD and related comorbidities challenging [71].

In our study, FGF23 levels were higher in children receiving HD than PD (3986.47 [518.99–7326.01] RU/mL vs. 1350.13 [583.91–5153.06] RU/mL, respectively). A single-center study with a small number of pediatric patients on dialysis reported no significant differences between patients undergoing HD and PD (*p* = 0.772) [72]. In adult patients, Bi et al. (2017) showed higher FGF23 levels for patients on HD compared with PD [70, 77]. However, our study results did not show the difference in the FGF23 levels between dialysis modalities, likely due to our small sample size.

The preference for PD over HD is well-established [73, 74]. Previous studies have shown that FGF23 is a biomarker for effective PD, showing associations with serum phosphate levels, residual kidney function (RKF), dialysis vintage, and renal phosphate clearance [75, 76]. Patients on PD with lower FGF23 levels have improved MBD control, sustained RKF, and higher PD adequacy measured by kT/v [76]. Due to the unavailability of automated PD (APD) machines, our PD patients undergo continuous ambulatory PD, despite superior outcomes with APD. In addition to being preferred for social reasons, APD provides better ultrafiltration, less edema, lower mean blood pressure, lower peritonitis rates, and fewer hospital admissions [77, 78]. The discrepancy in the numbers of patients receiving HD and PD may explain the lack of significance in our study, as patients may not have been effectively dialyzed. Another important point is that the PD adequacy test is not routinely performed in our center. Therefore, we could not assess whether inadequate dialysis contributed to our results. Furthermore, our patients on HD are limited to twice-a-week dialysis, rather than the recommended 3–4 times a week. Therefore, underdialysis may have influenced the non-significant results, particularly in the MBD parameters observed in our HD group.

Our study reported valuable new data on factors contributing to increased FGF23 and its association with CKD-MBD in children. Our study considered the contributions of nutritional status, MBD, CKD progression, and dialysis modalities. Understanding the role of FGF23 in CKD-MBD could help prevent further complications in affected individuals. We reported compelling data with notably high values, potentially influenced by late diagnoses and a high proportion of patients with impaired growth. This highlights the importance of early diagnosis and effective management and is an example of how challenges can be addressed in a severely uncontrolled pediatric population with CKD.

Limitations of our study include the relatively small number of patients, which hindered our ability to recognize significant clinical characteristics and clarify the roles of cIMT and FGF23. Another limitation is the potential underdosing of medications, particularly calcitriol, as it was not individually tailored according to laboratory parameters. Underdosing could have contributed to the high MBD rates in our study. Importantly, in a developing country with limited resources, routine laboratory check-ups are not feasible for some relevant biomarkers, including vitamin D levels. Government funding restricts the supply of calcitriol to 0.25 mcg thrice weekly for patients with a body weight of ≤ 30 kg and 0.5 mcg thrice weekly for those > 30 kg, limiting the ability to optimize dosing. No patients received other vitamin D supplementations during the study period. The absence of standard treatments like cinacalcet and intravenous calcitriol in Indonesia further constrained our exploration of appropriate management strategies for CKD-related comorbidities. Additionally, the lack of routine alkaline phosphatase (ALP) assessment in our study further restricted the comprehensive evaluation of CKD-MBD parameters. ALP and PTH are linked to bone turnover and mineralization, and ALP is an indirect biomarker for CKD [79–81]. These limitations underscore the challenges faced in resource-constrained settings, impacting the precision of our findings and emphasizing the need for cautious interpretation of our results. These limitations also prevent us from adhering to the CKD-MBD management guidelines, including recommended laboratory tests, therapeutic choices, and dosages [34]. Furthermore, cIMT measurements in our study were conducted once by a single examiner for each assessment, which were performed at different times rather than utilizing batch readings, as recommended for optimal consistency [57].

Finally, a limitation arises from the nature of our center as a regional referral center. For some patients, there may have been a relatively short time between first admission to our center and inclusion in our study. This may not accurately reflect actual CKD progression since individuals could have been living with the condition for an extended period before diagnosis. This discrepancy makes assessing the impact of FGF23 and cIMT levels on CKD-MBD challenging, as the full extent of disease progression may not have been captured. Furthermore, we do not have histopathological data for the glomerular disorders because kidney biopsies were not done in our center until late 2021. Therefore, we could not assess the correlations of cIMT, FGF23, and MBD with specific morphological changes in the kidneys.

A long-term prospective cohort study is needed in the future, particularly to address the hypotheses our current study did not support; for example, the association between high cIMT, high FGF23, and poor MBD control, involving all MBD parameters, as shown in Fig. 1. This future study should be conducted on a larger sample size with healthy controls and include serial testing of PTH and other MBD markers. Additionally, the standardized treatment protocol should be implemented to eliminate potential confounding factors.

## Conclusion

FGF23 levels increased as CKD progressed, and MBD was more prevalent in advanced kidney disease. High FGF23 levels potentially correlate with a greater MBD risk. The hypothesis that increased cIMT is influenced by abnormal BMI, hypertension, advanced CKD, presence of MBD, high FGF23, and high LVMI was not confirmed in this study. A more extensive study involving children with CKD is needed to confirm factors influencing cIMT and clarify the relationship between these factors, FGF23, and MBD.

## Electronic supplementary material

Below is the link to the electronic supplementary material.

Supplementary Material 1

## Data Availability

The datasets generated and analyzed during the current study are not publicly available but are available from the corresponding author on reasonable request.
