# Supplementary material for: Carotid intima-media thickness, fibroblast growth factor 23, and mineral bone disorder in children with chronic kidney disease
Source: BMC Nephrol. 2024 Oct 21;25:369. doi: 10.1186/s12882-024-03771-z (PMC11494757; doi:10.1186/s12882-024-03771-z)
Supplement: Supplementary file 1 — Supplementary Material 1 [file 12882_2024_3771_MOESM1_ESM.docx]

Supplementary Materials for:

**Carotid Intima-Media Thickness, Fibroblast Growth Factor 23, and**

**Mineral Bone Disorder in Children with Chronic Kidney Disease**

Retno Palupi-Baroto, Kristia Hermawan, Indah Kartika Murni, Tiara Nurlita, Yuli Prihastuti, Ira Puspitawati, Chika Carnation Tandri, Cahyani Gita Ambarsari

*Corresponding Author: retno.palupibaroto@ugm.ac.id

Table S1. Blood pressure categories and stages based on

the 2017 American Academy of Pediatrics Blood Pressure Clinical Practice Guideline[26]

| **Definition** | **Children Aged 1–13 years** | **Children Aged ≥ 13 years** |
| --- | --- | --- |
| Normal BP | < 90^th^ percentile | < 120/<80 mmHg |
| Elevated BP | ≥ 90^th^ percentile to < 95^th^ percentile or 120/80 mmHg to < 95th percentile (whichever is lower) | 120/< 80 to 129/< 80 mmHg |
| Stage 1 hypertension | ≥ 95^th^ percentile to < 95^th^ percentile +12 mmHg, or 130/80 to 139/89 mmHg (whichever is lower) | 130/80 to 139/89 mmHg |
| Stage 2 hypertension | ≥ 95^th^ percentile + 12 mmHg, or ≥ 140/90 mmHg (whichever is lower) | ≥ 140/90 mmHg |

BP: blood pressure

Table S2. Normal ranges for serum phosphorus and total calcium[38]

| **Age (years)** | **Serum phosphorus (mg/dL)** | **Serum total calcium (mg/dL)** |
| --- | --- | --- |
| 0–0.25 | 4.8–7.4 | 8.8–11.3 |
| 1–5 | 4.5–6.5 | 9.4–10.8 |
| 6–12 | 3.6–5.8 | 9.4–10.3 |
| 13–20 | 2.3–4.5 | 8.8–10.2 |

Table S3. Target range of intact PTH by CKD stage[38]

| **CKD stage** | **GFR range (mL/min per 1.73 m^2^)** | **Target intact PTH (pg/mL)** |
| --- | --- | --- |
| 3 | 30–59 | 35­­–70 |
| 4 | 15–29 | 70–110 |
| 5 | < 15 or dialysis | 150–300 |

CKD: chronic kidney disease; GFR: glomerular filtration rate; PTH: parathyroid hormone

Table S4. Normal range for uric acid[39]

| **Gender** | **Age (years)** | **Uric acid (mg/dL)** |
| --- | --- | --- |
| Male | 1–10 | 2.4–5.4 |
|  | 11 | 2.7–5.9 |
|  | 12 | 3.1–6.4 |
|  | 13 | 3.4–6.9 |
|  | 14 | 3.7–7.4 |
|  | 15 | 4.0–7.8 |
|  | ≥16 | 3.7–8.0 |
| Female | 1 | 2.1–4.9 |
|  | 2 | 2.1–5.0 |
|  | 3 | 2.2–5.1 |
|  | 4 | 2.3–5.2 |
|  | 5 | 2.3–5.3 |
|  | 6 | 2.3–5.4 |
|  | 7–8 | 2.3–5.5 |
|  | 9–10 | 2.3–5.7 |
|  | 11 | 2.3–5.8 |
|  | 12 | 2.3–5.9 |
|  | ≥ 13 | 3.7–7.4 |

Table S5. Primary diagnosis of CKD

| **Diagnosis** | **Overall** | **CKD Stages** | | |
| --- | --- | --- | --- | --- |
|  |  | **Stages 2–3** | **Stages**  **4 and 5ND** | **Stage 5D** |
| Glomerular diseases | 20 |  |  |  |
| Nephrotic syndrome | 12 | 5 | 2 | 5 |
| Post-infectious glomerulonephritis | 6 | 2 | 0 | 4 |
| Lupus nephritis | 2 | 1 | 1 | 0 |
| CAKUT disorders | 18 |  |  |  |
| Obstructive uropathy | 8 | 2 | 3 | 3 |
| Reflux nephropathy | 3 | 0 | 1 | 2 |
| Kidney aplasia/hypoplasia/dysplasia | 7 | 1 | 2 | 4 |
| Others | 4 |  |  |  |
| Polycystic kidney disease | 3 | 0 | 1 | 2 |
| Nephrocalcinosis due to Williams syndrome | 1 | 0 | 1 | 0 |

CAKUT: congenital anomalies of kidney and urinary tract; CKD: chronic kidney disease; CKD 5ND: eGFR < 15 mL/min per 1.73 m^2^ not on dialysis; CKD 5D: eGFR < 15 mL/min per 1.73 m^2^ with dialysis

Table S6. Baseline characteristics of the study population based on primary diagnosis of CKD

| **Characteristics** | **CKD Stages** | | |
| --- | --- | --- | --- |
|  | **Glomerular Diseases** | **CAKUT Disorders** | **Others** |
| Number of patients | 20 | 18 | 4 |
| Age of CKD diagnosis, years | 10.65  (8.15 – 13.75) | 11  (6.3 – 12.8) | 14.35  (12.5 – 16.1) |
| Age as a subject in our study, years | 11.66  (9.61 – 15.85) | 12.1  (8.02 – 14.98) | 14.73  (13.31 – 16.96) |
| Sex, female, n (%) | 9 (45) | 11 (61.11) | 2 (50) |
| Dialysis duration, months | 11.54  (9.33 – 25.08) | 17.92  (12.95 – 26.83) | 25.1  (3.62 – 46.58) |
| Height SDS* | -2.27  (-3.48 – -1.27) | -2.4  (-3.65 – -1.76) | -2.19  (-4.1 – -0.81) |
| BMI^†^, kg/m^2^ | 18.7  (16.48–23.53) | 14.58  (13.59 – 18.38) | 20.57  (15.17 – 25.43) |
| Anemia, n (%) | 15 (75) | 13 (72.22) | 4 (100) |
| Hypertension, n (%) | 11 (55) | 10 (55.56) | 1 (25) |
| Mineral bone disorders, n (%) | 13 (65) | 15 (83.33) | 4 (100) |
| Increased cIMT, n (%) | 3 (15) | 5 (27.78) | 1 (25) |

BMI: body mass index; CAKUT: congenital anomalies of the kidney and urinary tract; CKD: chronic kidney disease; N/A: not applicable; NS: not significant; SDS: standard deviation score

^*^Based on Centers for Disease Control and Prevention (CDC) Growth Chart[40]

^†^BMI: underweight (BMI-for-age < 5^th^ percentile), normal (BMI-for-age ≥ 5^th^ and < 85^th^ percentile), overweight (BMI-for-age ≥ 85^th^ and < 95^th^ percentile), obese (BMI-for-age ≥ 95^th^ percentile)[40]

^‡^Glomerular diseases: nephrotic syndrome, post-infectious glomerulonephritis, lupus nephritis

Table S7. FGF23 values based on CKD characteristics

| **Variables** | **Group (n)** | **FGF23 value (RU/mL) (median (IQR))** | **Group (n)** | **FGF23 value (RU/mL)**  **(median (IQR))** |
| --- | --- | --- | --- | --- |
| Sex | Male (20) | 827.52 (312.37–4107.48) | Female (22) | 723.56 (264.88–1079.4) |
| Stunted | Yes (29) | 604.83 (254.83–2984.87) | No (13) | 778.9 (461.69–1286.36) |
| Anemia | Yes (32) | 836.42 (405.1–3231.27) | No (10) | 312.51 (221.58–1008.86) |
| Hypertension | Yes (22) | 914.56 (484.38–5151.56) | No (20) | 415.8 (231.17–1401.99) |
| MBD | Yes (32) | 1055.58 (410.91–4107.48) | No (10) | 333.77 (221.58–341.43) |
| Increased cIMT | Yes (9) | 1079.4 (259.1–1493.2) | No (33) | 604.83 (297.63–1413.91) |
| Receiving KRT^*^ | Yes (20) | 1350.13 (583.91–6037.9) | No (22) | 365.02 (207.5–1008.86) |
| PD/HD as KRT modality | PD (16) | 1350.13 (583.91–5153.06) | HD (4) | 511.66 (264.88–1286.36) |

CKD: chronic kidney disease; FGF23: fibroblast growth factor 23; HD: hemodialysis; IQR: interquartile range; KRT: kidney replacement therapy; MBD: mineral bone disease; PD: peritoneal dialysis

^*^KRT refers to HD and PD

Table S8. Plasma FGF23 and bone mineral status in patients with CKD stage 5

receiving dialysis

| **Characteristics** | **Hemodialysis**  **(n = 4)** | **Peritoneal Dialysis**  **(n = 16)** |
| --- | --- | --- |
| Calcium (mg/dL) | 9.52 (7.52–10.4) | 8.98 (7.88–10.56) |
| Phosphate (mg/dL) | 4.4 (2.65–5.85) | 3.65 (2.65–4.65) |
| Ca x P product (mg^2^/dL^2^) | 36.93 (21.25–55.79) | 31.92 (23.64–51.32) |
| Albumin (g/dL) | 3.84 (3.49–3.95) | 3.3 (2.94–4.03) |
| Corrected Calcium (mg/dL) | 9.56 (7.84–10.53) | 9.51 (8.29–11.2) |
| FGF23 (RU/mL) | 3986.47 (518.99–7326.01) | 1350.13 (583.91–5153.06) |
| PTH (pg/mol)^*^ | 478^†^ | 221.2 (87.7–450.3) |
| Uric acid (mg/dL)^‡^ | 6.5 (3.6–7.9) | 4.9 (4.1–5.6) |
| cIMT (mm) | 0.49 (0.4–0.52) | 0.42 (0.4–0.46) |
| LVMI (g/m^2^) | 129.35 (83.41–144.98) | 97.12 (55.84–153.07) |

Data are stated in median (IQR)

Ca: calcium; cIMT: carotid intima-media thickness; CKD: chronic kidney disease; FGF23: fibroblast growth factor 23; LVMI: left ventricular mass index; P: phosphate; PTH: parathyroid hormone

^*^PTH levels data from 9 subjects; ^†^PTH level data from only 1 subject; ^‡^Uric acid levels data from 17 subjects.

Table S9. Plasma FGF23 and cIMT according to hypertension stages^*^

| **Characteristics** | **Hypertension Stage** | | | | **p value** |
| --- | --- | --- | --- | --- | --- |
|  | **Normal BP**  **(n = 14)** | **Elevated BP (n = 6)** | **Stage 1 HTN**  **(n = 8)** | **Stage 2 HTN**  **(n = 14)** |  |
| cIMT, mm | 0.42 (0.4–0.46) | 0.42 (0.41–0.43) | 0.4 (0.38–0.46) | 0.42 (0.4–0.5) | 0.81 |
| Normal BP vs. elevated BP |  | | | | 0.34 |
| Normal BP vs. stage 1 HTN |  | | | | 0.24 |
| Normal BP vs. stage 2 HTN |  | | | | 0.42 |
| Elevated BP vs. stage 1 HTN |  | | | | 0.41 |
| Elevated BP vs. stage 2 HTN |  | | | | 0.29 |
| Stage 1 HTN vs. stage 2 HTN |  | | | | 0.19 |
| Plasma FGF23, RU/mL | 312.51 (171.79–1008.86) | 1142.01 (461.69–3477.66) | 501.37 (212.76–914.56) | 1182.88 (563–6921.24) | 0.02 |
| Normal BP vs. elevated BP |  | | | | 0.04 |
| Normal BP vs. stage 1 HTN |  | | | | 0.39 |
| Normal BP vs. stage 2 HTN |  | | | | 0.002 |
| Elevated BP vs. stage 1 HTN |  | | | | 0.09 |
| Elevated BP vs. stage 2 HTN |  | | | | 0.3 |
| Stage 1 HTN vs. stage 2 HTN |  | | | | 0.01 |

Data are stated in median (IQR)

BP: blood pressure; cIMT: carotid intima-media thickness; FGF23: fibroblast growth factor 23; HTN: hypertension

^*^Kruskal Wallis and Dunn’s test
